# Dichotomous Roles of *Men1* in Macrophages and Fibroblasts in Bleomycin—Induced Pulmonary Fibrosis

**DOI:** 10.3390/ijms23105385

**Published:** 2022-05-11

**Authors:** Yuanhua Lu, Jianan Zhao, Yafei Tian, Dan Shao, Zhiqi Zhang, Siqi Li, Jialin Li, Hugang Zhang, Wei Wang, Ping Jiao, Jie Ma

**Affiliations:** School of Pharmaceutical Sciences, Jilin University, Changchun 130021, China; luyuanhua91@163.com (Y.L.); taboonan1992@163.com (J.Z.); tianyf19@mails.jlu.edu.cn (Y.T.); shaodan21@mails.jlu.edu.cn (D.S.); zhiqiz21@mails.jlu.edu.cn (Z.Z.); siqil21@mails.jlu.edu.cn (S.L.); jialinli_@hotmail.com (J.L.); zhanghg9901@163.com (H.Z.); boheng2010@126.com (W.W.)

**Keywords:** *Men1*, pulmonary fibrosis, macrophage, pyroptosis, OPN

## Abstract

Pulmonary fibrosis therapy is limited by the unclear mechanism of its pathogenesis. C57BL/6 mice were used to construct the pulmonary fibrosis model in this study. The results showed that *Men1*, which encodes menin protein, was significantly downregulated in bleomycin (BLM)—induced pulmonary fibrosis. Mice were made to overexpress or had *Men1* knockdown with adeno-associated virus (AAV) infection and then induced with pulmonary fibrosis. BLM—induced pulmonary fibrosis was attenuated by *Men1* overexpression and exacerbated by *Men1* knockdown. Further analysis revealed the distinct roles of *Men1* in fibroblasts and macrophages. *Men1* inhibited fibroblast activation and extracellular matrix (ECM) protein expression while promoting macrophages to be profibrotic (M2) phenotype and enhancing their migration. Accordingly, pyroptosis was potentiated by *Men1* in mouse peritoneal macrophages (PMCs) and lung tissues upon BLM stimulation. Furthermore, the expression of profibrotic factor OPN was positively regulated by menin in Raw264.7 cells and lung tissues by binding to the *OPN* promoter region. Taken together, although *Men1* showed antifibrotic properties in BLM—induced pulmonary fibrosis mice, conflictive roles of *Men1* were displayed in fibroblasts and macrophages. The profibrotic role of *Men1* in macrophages may occur via the regulation of macrophage pyroptosis and OPN expression. This study extends the current pathogenic understanding of pulmonary fibrosis.

## 1. Introduction

Pulmonary fibrosis, an irreversible interstitial lung disease, has a complex and largely unclear underlying mechanism that limits its therapeutic options [1]. Hence, it is vital to explore the detailed mechanism of pulmonary fibrosis to uncover related therapeutic targets.

The development of pulmonary fibrosis involves multiple cell types such as neutrophils, macrophages, T cells, fibroblasts, epithelial cells, and endothelial cells. Macrophages, the pivotal regulator of lung disease, play a crucial role in the progression of pulmonary fibrosis [2]. Upon epithelial damage, macrophages secrete chemokines, inflammatory mediators, and matrix metalloproteinases to drive the damage response. Macrophage removal after injury greatly diminishes the inflammatory response with damage repair and regeneration [3,4]. Thereafter, in subsidence of inflammation, macrophages produce growth factors and soluble mediators to promote fibroblasts proliferation, activation, and blood vessel development [5,6,7]. Subsequently, macrophages acquire the anti-inflammatory phenotype, known as the alternative activated M2 phenotype, and secrete anti-inflammatory mediators such as IL-10 and TGF-β to suppress inflammatory response and facilitate wound closure [8]. Therefore, the M2 phenotype of macrophages is recognized as profibrotic. Dysfunction of macrophages during the damage and repair process can lead to aberrant repair and, most probably, fibrosis [9,10,11].

Irritants such as bleomycin (BLM), silica, and asbestos can induce inflammasomes in macrophages. Studies have shown that NLRP3 inflammasome is essential for the development of pulmonary fibrosis [12,13,14,15]. Inflammasomes sense the stimulus and activate adaptor ASC binding to pro-Caspase1. The cleaved Caspase1 then activates pro-IL-1β and pro-IL-18 with concurrent cleavage of GSDMD to form pores in the cytoplasmic membrane, which ultimately leads to pyroptosis, one of the programmed necrosis [16]. Liang et al. showed that inhibition of NLRP3 inflammasome activation and pyroptosis ameliorated BLM−induced pulmonary fibrosis in mice [17]. In BLM—induced mice, *Nlrp3*^−/−^, *Asc*^−/−^, and *Casp1*^−/−^ mice exhibited the abrogation of pulmonary inflammation and fibrosis, which highlights the essential role of pyroptosis in the development of pulmonary fibrosis [18]. Pyroptosis may be the potential mechanism of macrophages inducing pulmonary fibrosis.

Osteopontin (OPN), primarily produced by macrophages, is crucial for several pathophysiological processes, including bone resorption, malignant transformation, inflammation, and tissue repair [19,20]. Notably, Takahashi and colleagues found that OPN was overexpressed in alveolar macrophages of the BLM—induced pulmonary fibrosis mice [21]. Interestingly, in BLM—induced mice models, *OPN* knockout alleviated lung fibrosis [22]. A recent study in idiopathic pulmonary fibrosis (IPF) patients showed the prime contribution of OPN^hi^ macrophages in lung fibrosis [23]. Meanwhile, OPN secreted by eosinophils greatly facilitated airway fibrosis [24]. Therefore, OPN, especially of macrophage origin, plays a critical role in the progression of pulmonary fibrosis.

The *Men1* gene, which gets mutated in multiple endocrine neoplasia type 1 (MEN1) syndrome patients, encodes the scaffold protein named menin [25]. Menin possesses diverse functions, generally by regulating gene expression via transcriptional activators such as Runx2, c-Myc, and histone modifiers or transcriptional repressors such as NF-κB and JunD [26,27,28,29,30]. It is an important regulator of tumors and sometimes plays dichotomous functions. Menin has been linked to leukemogenesis and the progression of liver cancer [31,32], while it plays a suppressive role in lung, breast, and prostate cancers [33,34,35]. Notably, some studies also reported the profibrotic role of the *Men1* gene in liver fibrosis [36,37]. Interestingly, like the dichotomous roles in cancer, *Men1* also displayed opposite roles in fibrogenesis in different organs. For instance, Wei and colleagues found that *Men1* played an antifibrotic role in radiation—induced pulmonary fibrosis [38]. However, direct in vivo evidence and detailed mechanisms were lacking to explain the role of *Men1* in pulmonary fibrogenesis. Considering the important role of *Men1* in lung cancer [39,40] and the close relationship between pulmonary fibrosis and lung cancer (pulmonary fibrosis is a risk factor for lung cancer, and both have common pathogenic mechanisms [41]), studying the detailed function of *Men1* in pulmonary fibrogenesis appears important.

This study examined the close relationship between *Men1* and pulmonary fibrosis and uncovered the dichotomous roles of *Men1* during BLM—induced fibrogenesis, highlighting the novel mechanism of pulmonary fibrosis and the new function of *Men1* in the lung.

## 2. Results

### 2.1. Menin Was Downregulated in BLM—Induced Pulmonary Fibrosis

**Figure 1:**
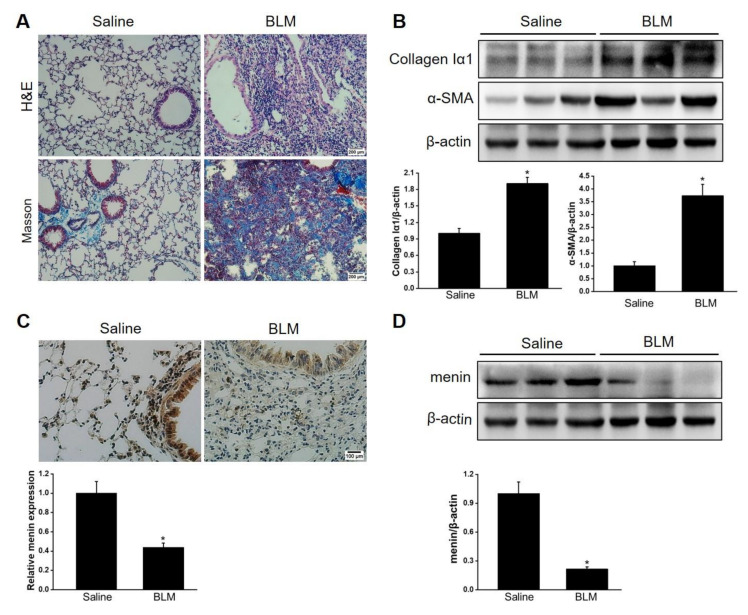
Menin was downregulated in BLM—induced pulmonary fibrosis. (**A**) H&E staining and Masson staining for lung tissues. Scale bar: 200 μm. (**B**) The protein levels of collagen Iα1 and α-SMA were determined by Western blotting. The blots were quantified by Image J (*n* = 3 for each group). (**C**) IHC staining was performed to measure the expression and distribution of menin in lung tissues and quantified by Image J software (*n* = 3 for each group). Scale bar: 100 μm. (**D**) The expression of menin was detected by Western blotting and quantified by Image J software (*n* = 3 for each group). * *p* < 0.05.

BLM is one of the most common reagents used to induce pulmonary fibrosis [42]. This study constructed BLM—induced pulmonary fibrosis mice models as described previously [43]. The lung showed structural disorder with a fibrotic scar and abundant collagen deposition after BLM stimulation for 21 days (Figure 1A). Furthermore, collagen Iα1, the main content of extracellular matrix (ECM), and α-SMA, a marker of activated fibroblast, were significantly upregulated in BLM-stimulated lung tissue compared with healthy lung tissue (Figure 1B). Overall, these results indicated that the mice suffered from pulmonary fibrosis after BLM stimulation. IHC staining revealed the abundant distribution of menin in mice lung tissue (Figure 1C). Notably, menin expression was dramatically lower in BLM-stimulated lung tissue compared with that in normal lung tissue, especially in the fibrotic lesions (Figure 1C,D). Therefore, we suspected that menin played a crucial role in the development of BLM—induced pulmonary fibrosis.

### 2.2. Men1 Overexpression Improves BLM—Induced Pulmonary Fibrosis

**Figure 2:**
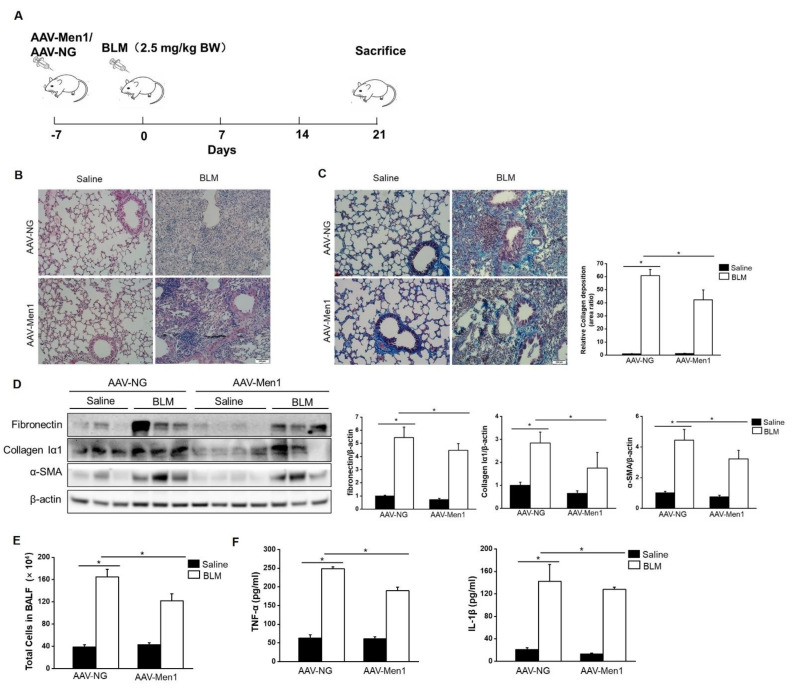
*Men1* overexpression inhibited BLM—induced pulmonary fibrogenesis. (**A**) Schematic showing the procedure of animal experiments. (**B**) H&E staining of lung tissues. Scale bar: 200 μm. (**C**) Masson staining of lung tissues. Collagen deposition was quantified by Image J software. Scale bar: 200 μm. (**D**) The protein levels of fibronectin, collagen Iα1, and α-SMA in lung tissue were determined by Western blotting, and quantification was performed by Image J software. (**E**) Total cells of BALF were counted by hemocytometer. (**F**) Contents of TNF-α and IL-1β in BALF were estimated by ELISA. *n* = 6 for Saline groups and *n* = 7 for BLM groups. * *p* < 0.05.

To examine the role of menin in pulmonary fibrogenesis, AAV-Men1 or AAV-NG virus were delivered to the mice lung via nasal inhalation. The mice were then induced pulmonary fibrosis by intra-tracheal injection of BLM (Figure 2A). The expression and distribution of menin were determined. A > 3-fold increase in menin expression was detected in the lung of AAV-Men1 mice compared with that of AAV-NG mice (Figure S1A,B). Accordingly, menin was downregulated upon BLM stimulation both in AAV-NG and AAV-Men1 mice compared to the control mice (saline-treated) (Figure S1A,B). Fibrotic lesions and the expression level of ECM proteins and α-SMA were quantified by histological staining. Figure 2B shows the presence of an obvious fibrotic area upon BLM-induction, while the fibrotic area in BLM-treated AAV-Men1 mice was smaller than that in AAV-NG mice. Furthermore, collagen deposition in BLM—induced AAV-Men1 mice was significantly less compared to that in BLM—induced AAV-NG mice (Figure 2C). Moreover, the BLM—induced increment in α-SMA, collagen Iα1, and fibronectin expression in AAV-Men1 mice was significantly less than that in AAV-NG mice (Figure 2D).

An increase in total cell numbers and enrichment of inflammatory cytokines (TNF-α and IL-1β) in BALF are the hallmarks of BLM—induced pulmonary fibrosis [44,45]. Accordingly, the same was observed upon BLM stimulation (Figure 2E,F). However, *Men1* overexpression suppressed cells and inflammatory cytokines enrichment in BLAF (Figure 2E,F). In all, *Men1* overexpression relieved BLM—induced pulmonary fibrosis.

### 2.3. Inhibition of Men1 Expression Exacerbates BLM—Induced Pulmonary Fibrosis

**Figure 3:**
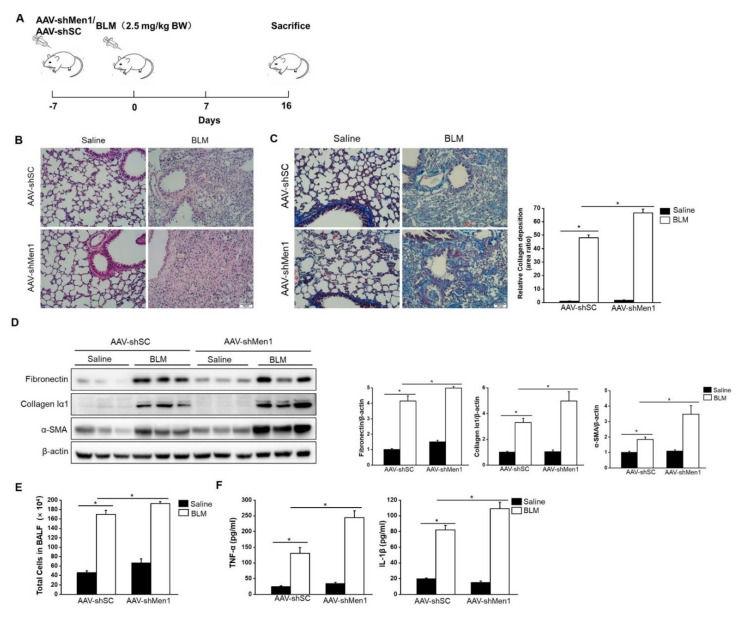
BLM—induced pulmonary fibrosis was worsened by *Men1* knockdown. (**A**) Timeline for AAV infection and BLM stimulation in mice. (**B**) Morphology of lung tissue was examined by H&E staining. Scale bar: 200 μm. (**C**) Masson staining of lung tissue. Collagen deposition was quantified by Image J software. Scale bar: 200 μm. (**D**) Western blotting was used to estimate the protein levels of fibronectin, collagen Iα1, and α-SMA in lung tissue. Data were quantified by Image J software. (**E**) The total number of cells was counted in BALF. (**F**) The contents of TNF-α and IL-1β were estimated by ELISA. *n* = 5 for each group. * *p* < 0.05.

To further validate the antifibrotic role of menin, mice were infected with the AAV-shMen1 virus to knock down *Men1* expression and then exposed to BLM to induce pulmonary fibrosis (Figure 3A). The expression of menin in lung tissue was inhibited by ~50% upon AAV-shMen1 virus infection compared to AAV-shSC virus infection (Figure S2). Importantly, lowered *Men1* expression indeed exacerbated BLM—induced pulmonary fibrosis as the scar area further expanded and collagen deposition increased in AAV-shMen1 mice compared to that in AAV-shSC mice upon BLM treatment (Figure 3B,C). Furthermore, the levels of α-SMA, fibronectin, and collagen Iα1 further increased after *Men1* knockdown upon BLM stimulation (Figure 3D). Meanwhile, IF data showed that most cells in fibrotic lung interstitial tissue, in which fibroblasts locate, expressed α-SMA upon BLM treatment, while cells of healthy mice rarely expressed α-SMA. This indicated that the increment in α-SMA was mostly from the activated fibroblasts. However, the increment in α-SMA was suppressed in the AAV-shMen1 mice (Figure S2C). Additionally, the total cell numbers and contents of IL-1β and TNF-α in BALF increased in AAV-shMen1 mice compared with that in AAV-shSC mice after BLM stimulation (Figure 3E,F). Thus, inhibiting *Men1* expression worsened the BLM—induced pulmonary fibrosis.

### 2.4. The Antifibrotic Role of Men1 in Fibroblasts

**Figure 4:**
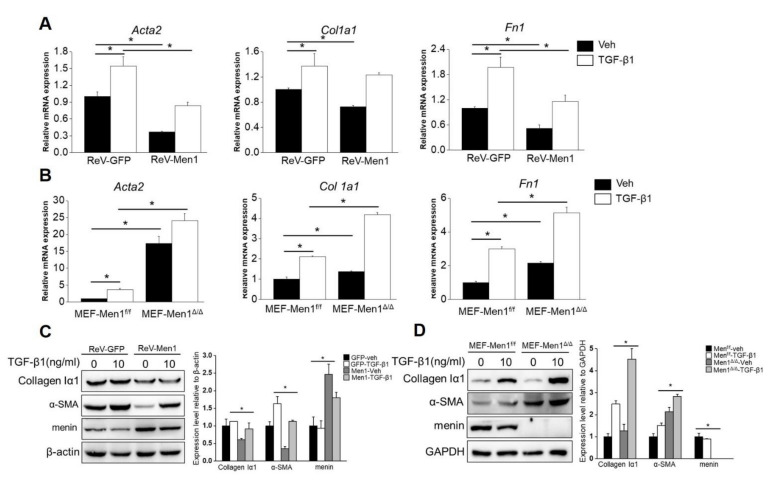
*Men1* suppresses fibroblast activation and ECM protein expression. (**A**) MEFs were infected with retrovirus to overexpress *Men1* and then exposed to recombinant TGF-β1; the expression of *Acta2, Col 1a1, and Fn1* were analyzed by qRT-PCR. (**B**) Men1-knockout MEFs and the control cells were treated with recombinant TGF-β1, and qRT-PCR was used to determine the expressions of *Acta2, Col 1a1, and Fn1.* Data in (**A**,**B**) are from three repeated experiments. (**C**,**D**) *Men1* overexpressing or knockout MEFs were stimulated with recombinant TGF-β1. The expression of ECM proteins was determined by Western blotting, blots from two repeated experiments were quantified by Image J software. * *p* < 0.05.

Fibroblast activation and excessive secretion of ECM directly contribute to pulmonary fibrosis [46]. Concerning the antifibrotic role of *Men1*, we determined its function in fibroblasts in vitro. The gene expression levels of *Acta2, Col 1a1*, and *Fn1*, which encode α-SMA, collagen Iα1, and fibronectin, respectively, were significantly decreased upon *Men1* overexpression and increased upon *Men1* knockout in MEF compared to that in control cells with or without TGF-β1 treatment, which could activate fibroblast (Figure 4A,B). Moreover, a significant decrease in Collagen Iα1 and α-SMA protein levels was observed after *Men1* overexpression in MEF (Figure 4C), while their levels were significantly higher in MEF-Men1^Δ/^^Δ^ cells than in MEF-Men1^f/^^f^ cells (Figure 4D). Thus, *Men1* probably inhibits fibrosis via suppressing fibroblast activation and secretion of ECM proteins.

### 2.5. Men1 Has a Profibrotic Role in Macrophages

**Figure 5:**
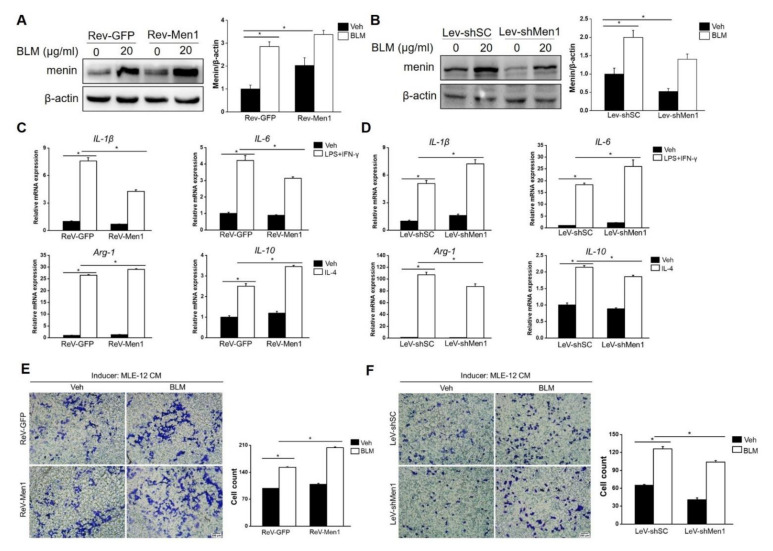
*Men1* plays a profibrotic role in macrophages. (**A**,**B**) Raw264.7 cells were infected with retrovirus (**A**) or lentivirus (**B**) to overexpress or knockdown *Men1*. The protein level of menin in Raw264.7 cells upon BLM treatment was determined by Western blotting, blots from three repeated experiments were quantified using Image J software (**C**,**D**). M1 macrophages were induced by LPS and IFNγ, and M2 macrophages were induced by mouse recombinant IL-4. The M1 (IL-1β and IL-6) and M2 (Arg-1 and IL-10) markers were determined by qRT-PCR. Data are from three repeated experiments. (**E**,**F**) MLE cells were exposed to BLM for 24 h, and the culture supernatant was used as a chemokine. Transwell inserts were used to detect the migration of of *Men1* overexpressing (**E**) or Knockdown (**F**) Raw264.7 cells upon the addition of supernatant from MLE cells. The statistics are from three repeated experiments. Scale bar: 200 μm. * *p* < 0.05.

Macrophages also play a critical role in the development of pulmonary fibrosis; unlike the direct action of fibroblasts in fibrosis, macrophages generally function as regulators [47]. Therefore, the role of *Men1* in macrophages upon BLM treatment was determined. Surprisingly, BLM stimulation dramatically upregulated the expression of menin in Raw264.7 cells, which was opposite to that observed in lung tissues of BLM−induced mice (Figure 5A,B). To further examine the function of *Men1* in macrophages, the expression of *Men1* was either upregulated (ReV-Men1 infection) or inhibited (LeV-shMen1 infection) using retro or lentivirus infection (Figure 5A,B). The alternative activated M2 phenotype is widely recognized as a profibrotic phenotype in macrophages. Notably, the Raw264.7 cells which overexpressed *Men1* or had inhibited *Men1* were induced to M1 or M2 phenotypes, respectively. Figure 5C,D show that LPS combined with IFN-γ stimulation remarkably upregulated *IL-1β* and *IL-6* expression, indicating the M1 phenotype in Raw264.7 cells. Importantly, overexpression of *Men1* downregulated *IL-1β* and *IL-6* while inhibiting *Men1* promoted these cytokines in M1 macrophages. In addition, the increment of *Arg-1* and *IL-10* levels suggested that Raw264.7 cells turned to the M2 phenotype upon IL-4 stimulation. Overexpression of *Men1* contributed to a further increase in the expression of *Arg-1* and *IL-10,* while inhibiting *Men1* lowered these cytokines in M2 macrophages (Figure 5C,D). Macrophages would migrate to the fibrotic lesion to facilitate fibrosis development. In this study, supernatant from BLM damaged MLE-12 cells induced migration in Raw264.7 cells, while *Men1* overexpressed in Raw264.7 cells improved their migration ability (Figure 5E). Contrarily, inhibiting *Men1* expression in Raw264.7 cells reduced their migration (Figure 5F). In all, *Men1* in macrophages facilitates fibrosis development via promoting M2 macrophage differentiation and macrophage migration, which is opposite to its function in fibroblasts.

### 2.6. Men1 in Macrophages Promotes the Profibrotic Activity of Fibroblast

**Figure 6:**
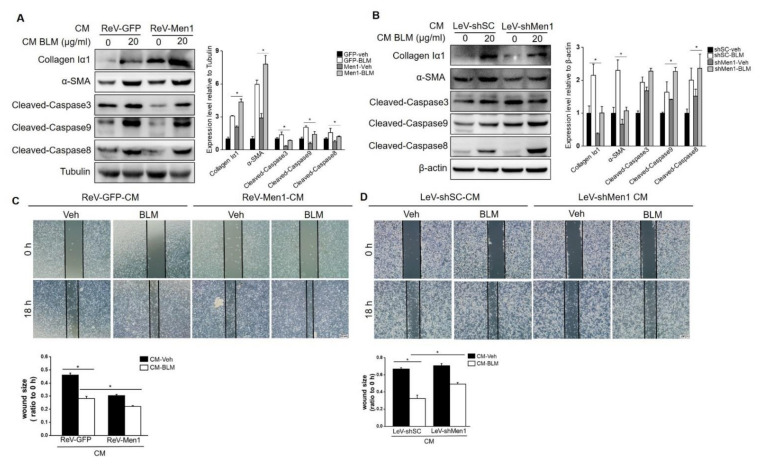
*Men1* in macrophages promotes MEF to the profibrotic phenotype. (**A**,**B**) Cell media (CM) from *Men1* overexpressing or *Men1* knockdown Raw264.7 cells with or without BLM treatment were added to MEF, and the protein levels of collagen Iα1, α-SMA, and cleaved Caspase3, 9, 8 were detected by Western blotting. Blots in two repeated experiments were analyzed by Image J software. (**C**,**D**) MEFs were plated into 12−well plates, and a scratch was made by a 100 μL sterile pipette tip. CM from Raw264.7 cells was added to MEF continuous culture. A total of three images for each group were photographed by an optical microscope, and the wound size from three repeated experiments was analyzed by Image J software. Scale bar: 200 μm. * *p* < 0.05.

As described above, *Men1* plays a conflictive role in fibroblasts and macrophages. To better understand this contradiction, we explored ways of *Men1* in macrophages to promote pulmonary fibrosis. Fibroblast activation and ECM secretion directly contribute to fibrosis, which is affected by macrophages [48]. In this study, CM from BLM treated or untreated Raw264.7 cells either overexpressing *Men1* or having inhibited *Men1* was collected to stimulate MEF. As shown in Figure 5A, CM from BLM treated Raw264.7 cells upregulated the expression of collagen Iα1 and α-SMA (Figure 6A,B). CM from *Men1* overexpressing Raw264.7 cells further increased α-SMA and collagen Iα1 levels, while CM from the Raw264.7 cells with inhibited *Men1* attenuated the levels of these markers (Figure 6A,B). Furthermore, overexpression of *Men1* in Raw264.7 relieved MEF apoptosis through CM treatment, and the opposite was observed if the expression of *Men1* was inhibited in Raw264.7 cells (Figure 6A,B). Moreover, fibroblast migration, which is critical to fibrosis development, was determined after CM treatment. Wound size became relatively smaller after BLM-CM stimulation, suggesting that BLM-stimulated macrophage could induce fibroblast migration via paracrine (Figure 6C,D). However, CM from *Men1* overexpressing Raw264.7 cells enhanced fibroblast migration (Figure 6C), while CM from *Men1* knockdownRaw264.7 cells diminished fibroblast migration (Figure 6D). Thus, *Men1* in macrophages can modify fibroblasts to the profibrotic phenotype facilitating fibrosis development.

### 2.7. Men1 Promotes Inflammasome Activation and Cell Pyroptosis upon BLM-Stimulation

**Figure 7:**
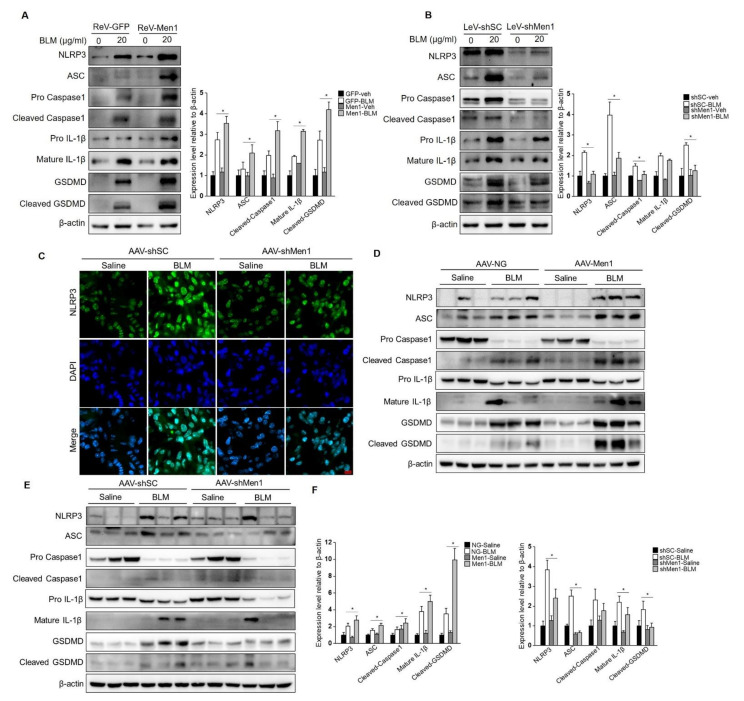
*Men1* promotes BLM—induced inflammasome activation and cell pyroptosis. (**A**,**B**) Raw264.7 cells either overexpressing or with *Men1* knockdown were exposed to 20 μM BLM, and the levels of pyroptotic pathway proteins were determined by Western blotting. Blots from two repeated experiments were quantified by Image J software. (**C**) The expression of NLRP3 in lung tissue was visualized with IF staining. NLRP3 and the nuclei were labeled with FITC and DAPI, respectively. Scale bar: 20 μm. (**D**,**E**) The levels of pyroptotic pathway proteins in lung tissue were determined by Western blotting. (**F**) Blots were quantified by Image J software, *n* = 6 for saline groups and *n* = 7 for BLM groups. * *p* < 0.05.

Further analyses were performed to explore the potential mechanism of menin function in macrophages during fibrogenesis. Since macrophage pyroptosis occurs during fibrosis and inflammasome activation in macrophages contributes to fibrosis development [17], we explored the role of *Men1* in BLM—induced pyroptosis in PMCs. As shown in Figure 7A,B, BLM treatment remarkably upregulated the level of inflammasome protein NLRP3, followed by the activation of the pyroptotic pathway, which is characterized by an increase in expression of ASC, Pro Caspase1, Pro IL-1β, and GSDMD. This also increased the levels of activated cleaved Caspase1, Mature IL-1β, and cleaved GSDMD. Importantly, overexpression of *Men1* enhanced the activation of the pyroptotic pathway, and inhibition of *Men1* attenuated BLM—induced Raw264.7 cells pyroptosis (Figure 7A,B). 

To explore this phenomenon in vivo, the inflammasomes were examined in the lung tissue of mice. BLM stimulation activated NLRP3 inflammasome in lung tissue while knocking down *Men1* attenuated them (Figure 7C). Consistently, BLM stimulation upregulated the expression and activation levels of proteins of the pyroptotic pathway in the total protein fraction of lung tissue. Overexpression of *Men1* further promoted the pyroptotic pathway, while knocking down *Men1* inhibited the same (Figure 7D,E). 

Although *Men1* improved BLM—induced pulmonary fibrosis in mice, the pathological process closely related to macrophages, such as pyroptosis, was still promoted by *Men1* expression.

### 2.8. Men1 Transcriptionally Promotes OPN Expression upon BLM Administration

**Figure 8:**
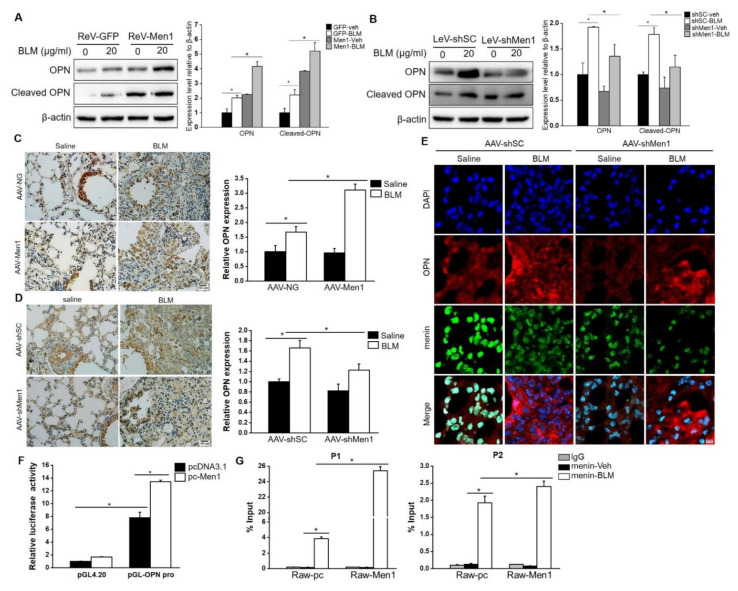
Menin positively regulates OPN expression at the transcription level. (**A**,**B**) Raw264.7 cells overexpressing *Men1* or having *Men1* knockdown were exposed to BLM, and the protein level of OPN was determined by Western blotting. Blots from two repeated experiments were quantified by Image J software. (**C**,**D**) IHC staining was performed to detect the expression and distribution of OPN in lung tissue of *Men1* overexpressing or *Men1* knockdown mice. Quantification of OPN expression was analyzed by Image J software (*n* = 6 for (**C**) and *n* = 5 for (**D**)). Scale bar: 100 μm. (**E**) The expressions and distributions of OPN and menin in lung tissues were determined by IF staining. Scale bar: 20 μm. (**F**) Dual−luciferase assay was performed in *Men1* overexpressing 293T cells which were transfected with pGL4.20 or pGL-OPN pro plasmids. The Firefly luciferase activity or Ranilla luciferase activity were determined by a luminescent microplate reader. Firefly luciferase activity was normalized to Ranilla luciferase activity. Data are from three repeated experiments (**G**). The binding of menin to OPN promoter was determined by ChIP assay in Raw264.7 cells which were treated with or without BLM. Chromatin fragments were precipitated with either anti-menin or anti-rabbit IgG antibodies. Specific primers were designed in the-2000 to 0 bp region of the OPN promoter. qRT-PCR was used to determine the enrichment of menin on the OPN promoter. Data are representative of three experiments. * *p* < 0.05.

OPN is an acknowledged profibrotic factor that is mostly produced by macrophages [49,50]. To further elucidate the possible profibrotic mechanism of *Men1* in macrophages, we explored its interaction with OPN. Results show that BLM stimulation significantly elevated the expression of OPN in Raw264.7 cells (Figure 8A,B). *Men1* overexpression further enhanced the expression of OPN (Figure 8A). Accordingly, the expression level of OPN was markedly decreased in the LeV-shMen1 group compared with that in the LeV-shSC group after BLM treatment (Figure 8B). The positive regulation of *Men1* to OPN is consistent with its profibrotic role in macrophages. In lung tissue of mice, the expression and distribution of OPN were elevated upon BLM stimulation. Notably, overexpression of *Men1* significantly promoted the OPN expression (Figure 8C), while the same was decreased in AAV-shMen1 mice compared to that in AAV-shSC mice upon BLM stimulation (Figure 8D). Specifically, the secreted OPN in lung tissue diminished in AAV-shMen1 mice compared to that in AAV-shSC mice, as shown by IF staining (Figure 8E). The positive regulation of *Men1* to OPN was further validated in the total protein samples of lung tissue (Figure S3A,B).

In general, menin regulates gene expression by binding to a promoter in a sequence non−specific manner [51]. Therefore, a dual-luciferase reporter gene assay was performed to investigate the OPN transcriptional regulatory role of menin. Data showed that the luciferase activity in pGL-OPN pro plasmid transfected cells was almost 8-fold higher than that in pGL4.20 plasmid transfected cells. Of note, *Men1* overexpression significantly enhanced the luciferase activity of the OPN promoter (Figure 8F), suggesting that menin regulated OPN expression in a transcriptional manner. ChIP assay was performed, and specific primers were designed to determine the interaction of menin with the OPN promoter (Figure S3C). We found significant enrichment of menin on the OPN promoter after BLM stimulation at P1 and P2 sites, and *Men1* overexpression further enhanced its binding (Figure 8G). Thus, menin binds to the OPN promoter to activate its transcription upon BLM stimulation.

## 3. Discussion

Menin is involved in various physiological and pathological processes [25]. This study showed that the expression of menin in lung tissue was remarkably downregulated in BLM—induced pulmonary fibrotic mice, which suggested a potential role of menin in lung fibrogenesis. Menin was first linked to liver fibrosis affecting hepatic stellate cell activation, which enhances liver fibrosis [36,37]. Wei and colleagues showed that menin expression was related to radiation—induced pulmonary fibrosis and inhibited fibroblast activation [38]. Our data verified the antifibrotic role of menin in BLM—induced pulmonary fibrosis mice. Our in vitro data showed that menin inhibited activation of MEF and directly affected the expression of ECM proteins; however, it modified Raw264.7 cells to profibrotic phenotype via promoting MEF to facilitate fibrogenesis indirectly. This study explored the function of menin during pulmonary fibrogenesis in vivo and in vitro, revealing its dichotomous roles in fibroblasts and macrophages.

A significant decrease in menin expression in fibrotic tissue strongly indicated the role of menin in pulmonary fibrogenesis, which is similar to its mode of action in lung cancer. *Men1* is an anti−oncogene in lung cancer and is expressed in low amounts in lung cancer tissues [33]. Accordingly, this study revealed that *Men1* is an antifibrotic gene in BLM—induced pulmonary fibrosis. Although Wei et al. proposed the antifibrotic role of *Men1* in radiation—induced pulmonary fibrosis, they did not prove the function of *Men1* in pulmonary fibrogenesis directly. In this study, *Men1* was overexpressed or knocked down in mice before the animal was exposed to BLM and verified a direct inhibitory role of *Men1* in pulmonary fibrogenesis. Pulmonary fibrosis is usually closely related to lung cancer. Since pulmonary fibrosis is a risk factor for lung cancer, many lung cancer therapies (BLM, radiation) may contribute to pulmonary fibrosis [41]. Thus, *Men1* would be a potential target in both primary diseases of the lung: lung cancer and pulmonary fibrosis. However, the mode of action of *Men1* needs to be revealed.

Multiple types of cells contribute to lung fibrogenesis. Fibroblasts function as the executor, while the other cells play the regulatory role [52]. In this study, BLM showed an opposite effect on menin expression in lung tissue and Raw264.7 cells. In lung tissue, the expression of menin was inhibited by BLM, while the opposite was observed in Raw264.7 cells. Previous studies reported that menin functions in a tissue−specific manner playing dichotomous functions in different tissues or different stages of the disease [25,26,53]. Accordingly, we speculated that menin plays different roles in different cell types during lung fibrogenesis. In support of this notion, we found that expressions of α-SMA, collagen Iα1, and fibronectin were significantly inhibited upon *Men1* overexpression and upregulated after *Men1* knockout in MEF, while *Men1* overexpression in Raw264.7 cells promoted cells differentiation to M2 phenotype. Raw264.7 cells migration induced by the supernatant from damaged epithelial cells was enhanced due to *Men1* overexpression, which was inhibited by *Men1* knockdown. These data preliminarily demonstrate different roles of menin in macrophage and fibroblast. Moreover, CM from Raw264.7 cells could activate MEF and promote its migration; also, overexpression of *Men1* in Raw264.7 cells potentiated these effects, while the *Men1* knockdown attenuated them. This highlights the opposite roles of menin in fibroblast and macrophage.

The potential mechanism of menin in macrophages during the development of pulmonary fibrosis was further investigated. Predominant cell pyroptosis was observed in fibrotic lung tissue and BLM stimulated macrophages. This coincides with previous studies that inflammasome activation along with cell pyroptosis contributes to fibrogenesis. Notably, altered *Men1* expression in lung tissue and macrophage significantly impacts cell pyroptosis. This was manifested as overexpression of *Men1* further promoted NLRP3 inflammasome activation and pyroptotic pathway, while the same was attenuated by *Men1* knockdown. Pyroptosis is distinguished from other cell deaths by membrane perforation and the release of inflammatory cytokines. IL-1β, released by pyroptotic cells, plays a critical role in fibrogenesis [54]. In this study, *Men1* indeed promoted IL-1β expression upon BLM stimulation in macrophages (data not shown), which is consistent with the above notion. Thus, promoting inflammasome activation and cell pyroptosis upon BLM treatment could be a potential mechanism of menin to play a profibrotic role in macrophages.

In the search for key mediators of *Men1* promoting fibrosis, menin interaction with OPN was investigated. As expected, OPN was upregulated upon BLM treatment both in mice and macrophages. Furthermore, *Men1* potentiated OPN expression under BLM stimulation. The results of the dual-luciferase reporter gene assay and ChIP suggested that menin transcriptionally upregulated OPN expression by binding to its promoter regions. Although further research is needed to explore the menin-OPN-lung fibrosis axis. Our data provide significant evidence that OPN may be a critical mediator of menin in macrophages to regulate lung fibrogenesis.

There are contrary views that cell pyroptosis and OPN expression were enhanced by *Men1* overexpression and suppressed by *Men1* knockdown, but BLM—induced fibrosis in the lung was attenuated upon *Men1* overexpression and deteriorated after *Men1* knockdown. The IHC results showed that after AAV infection, the distribution of menin in lung parenchyma was altered (Figures S1 and S2). Besides, fibroblasts, the main cell type in the lung parenchyma, are the executors of fibrogenesis. Menin in fibroblasts may play a key role during BLM—induced pulmonary fibrosis. However, pyroptosis and OPN are closely related to macrophages. *Men1* in local macrophages may influence pyroptosis and OPN expression, which is inconsistent with the development of fibrosis. These assumptions must be validated in the pulmonary fibrotic model of macrophage-specific knockout of *Men1*.

In summary, the present study demonstrates the antifibrotic role of menin in BLM−induced pulmonary fibrotic mice. The antifibrotic role of menin was supported by MEF, but the opposite function was observed in macrophages. Exacerbating cell pyroptosis and promoting OPN expression in macrophages could be vital mechanisms of menin in promoting BLM−induced fibrogenesis. This study revealed the dichotomous function of menin in different cell types which extends the pathogenic knowledge of pulmonary fibrosis.

## 4. Materials and Methods

### 4.1. Materials and Reagents

Plasmid pcDNA3.1 was purchased from Invitrogen Life Technology (Carlsbad, CA, USA); plasmids pLNCX2, pLNCX2-Men1, and pVSV-G for retrovirus packaging were gifts from Prof. Guanghui Jin from the Xiamen University; plasmids pGL4.20 and pGL4.74 were purchased from Promega (Madison, WI, USA). The Dulbecco’s Modified Eagle’s Medium (DMEM), Roswell Park Memorial Institute (RPMI) 1640 medium, and Transwell inserts were purchased from Corning (New York, NY, USA). The fetal bovine serum was obtained from Biological Industries (Kibbutz, Beit HaEmek, Israel). The adeno-associated virus (AAV), plasmids for lentivirus packaging, and Lenti-Easy Packaging System were purchased from Genechem (Shanghai, China). Bleomycin sulfate and LPS were obtained from Selleckchem (Houston, TX, USA) and Sigma-Aldrich (St. Louis, MO, USA), respectively. Recombinant mouse IL-4 and IFN-γ were obtained from Novus Biologicals (Centennial, CO, USA). FastStart Universal SYBR Green Master (ROX) reagent was purchased from Roche Diagnostics (Indianapolis, IN, USA). The Dual-Lumi luciferase reporter assay kit was obtained from Beyotime (Beijing, China). Chromatin Immunoprecipitation (ChIP) kit was purchased from Abcam (Cambridge, UK). The Immunohistochemistry (IHC) and DAB substrate kits were obtained from MXB biotechnology (Fuzhou, Fujian, China). The Masson’s Trichrome Stain kit was purchased from Solarbio (Beijing, China). Anti-menin, anti-NLRP3, and anti-Cleaved caspase3 antibodies were from Abcam (Cambridge, UK). Anti-Fibronectin, anti-α-SMA, anti-GSDMD, anti-F4/80, and anti-OPN antibodies were from Santa Cruz Biotechnology (Danvers, MA, USA). Anti-ASC, anti-Caspase1, anti-IL-1β, and anti-Cleaved caspase9 antibodies, FITC-conjugated goat anti-rabbit, and Cy3-conjugated goat anti-mouse secondary antibodies were obtained from Abclonal (Wuhan, Hubei, China). HRP-conjugated goat anti-mouse and goat anti-rabbit secondary antibodies, Alexa flour 488-conjugated goat anti-rat, and Alexa Flour 594 conjugated goat anti-rabbit secondary antibodies were purchased from Jackson ImmunoResearch laboratory (West Grove, PA, USA).

### 4.2. Animals

Male C57BL/6 mice (6–8 weeks old, 20–25 g), purchased from HFK Bioscience (Beijing, China), were housed in a temperature-controlled room under 12 h light and 12 h dark photocycle. Mice had ad libitum access to diet and water. All animal experiments were performed following the guidelines for the Care and Use of Experimental Animals of Jilin University and were approved by the Animal Experiment Ethics Committee of Jilin University.

To modulate the Men1 expression in the lung, mice were infected with AAV expressing *Men1* (pAAV-CMV-Men1-3xFLAG-P2A-mNeonGreen-tWPA, AAV-Men1) or shRNA targeting to *Men1* (pAAV-U6-shMen1-WPRE, AAV-shMen1) via nasal route. AAV-NG and AAV-shSC were used as respective controls. AAV diluted in 50 μL saline to 1.5 × 10^11^ v.g AAV-Men1 or 1.6 × 10^11^ v.g AAV-shMen1 was used as the infection dose.

### 4.3. Induction of Pulmonary Fibrosis Model

Pulmonary fibrosis models were established in mice after one week of AAV infection. Mice were anesthetized with 1% pentobarbital sodium and administrated with 2.5 mg/kg body weight of BLM in 50 μL normal saline using intratracheal injection. The control mice were injected with the same volume of normal saline. Mice in the AAV-Men1 and AAV-NG groups were sacrificed 21 days after BLM administration, while mice in the AAV-shMen1 and AAV-shSC groups were sacrificed 16 days after BLM injection, depending on the status of the mice. Bronchoalveolar lavage fluid (BALF) was collected via bronchoalveolar lavage with 0.6 mL of sterile PBS as described previously [55].

### 4.4. Histological Staining

The upper left lung lobe was removed, fixed in 4% paraformaldehyde for 48 h, and then embedded in paraffin. Embedded tissues were cut into 5 μm thick slices for histological staining. Tissue slices were deparaffinized with xylene, rehydrated with gradient alcohol, and then stained with Hematoxylin-eosin (H&E) or Masson trichrome reagents following the manufacturer’s instructions. For immunohistochemistry (IHC), tissue slices were immersed in 0.01 M sodium citrate and heated at 95 °C for 15 min for antigen retrieval, followed by incubation with 3% hydrogen peroxide at room temperature for 10 min to deactivate endogenous peroxidase; 5% BSA was used for blocking. Tissue sections were incubated with anti-menin (1:500) or anti-OPN (1:300) antibodies at 4 °C overnight. After PBS wash, sections were incubated with anti-rabbit (IHC kit) or anti-mouse (1:100) secondary antibodies at 37 °C for 1 h. A DAB substrate kit was used to visualize the staining. Histological images were photographed using a light microscope (Olympus, Tokyo, Japan), and the results were quantified with Image J software.

### 4.5. Immunofluorescence

The bottom left lung was removed and fixed in 4% paraformaldehyde for 48 h, embedded in the OCT compound, and then frozen. The frozen tissues were cut into 10 μm thick slices, fixed with cold acetone for 10 min, then incubated with 0.5% Triton X-100 for 30 min to permeate the cell membrane. Tissues were blocked with 2% goat serum and then incubated with anti-menin (1:300), anti-α-SMA (1:100), anti-F4/80 (1:100), anti-NLRP3 (1:100), anti-F4/80 (1:100) or anti-OPN (1:100) antibodies overnight at 4 °C. Next, tissue sections were incubated with fluorescence-conjugated secondary antibodies for 1 h at 37 °C in the dark. The cell nuclei were stained with DAPI. Fluorescent images were photographed with a laser scanning confocal microscope (Nikon, Tokyo, Japan).

### 4.6. Enzyme-Linked Immunosorbent Assay (ELISA)

The BALF contents of IL-1β and TNF-α were determined using the respective ELISA kit following the manufacturer’s instructions. Briefly, each well of the microplate was pre-coated with 100 μL of diluted capture antibodies overnight, followed by blocking with 1% BSA. Next, 100 μL sample or standard substance were added to each well and incubated for 2 h, followed by the addition of 100 μL detection antibodies per well. After that, samples were incubated with Streptavidin-HRP conjugated secondary antibodies for 30 min, followed by incubation with detection substrate. Lastly, 50 μL 2N H_2_SO_4_ was added to stop the reaction, and the sample absorbance was estimated using a microplate reader at 450 nm.

### 4.7. Cell Culture and Treatment

Murine Raw264.7 macrophages were obtained from the American Type Culture Collection (ATCC), and HEK-293T cells were purchased from Genechem (Shanghai, China). Murine alveolar epithelial cell line MLE-12 and immortalized murine embryo fibroblasts (MEF) were kindly provided by Prof. Guanghui Jin. All cells were grown in DMEM supplemented with 10% fetal bovine serum (FBS) and cultured at 37 °C and 5% CO_2_.

Raw264.7 cells were exposed to 20 μg/mL BLM for 48 h, and then the culture medium (CM) was subjected to centrifugation to remove the cell debris. The collected supernatant was treated with MEF. The M1 and M2 macrophages were induced as described previously [56].

### 4.8. Isolation of Mouse Peritoneal Macrophages (PMC)

PMCs were isolated as described previously [56]. Briefly, male C57BL/6 mice (8–10 weeks) were pre-treated with 1 ml 4% starch broth medium by intraperitoneal injection for 3 days consecutively. After 24 h of the last injection, mice were sacrificed and sterilized with 75% ethanol. The mice peritoneal cavity was washed with cold, sterile PBS, and the collected PBS wash was centrifuged at 300× *g* for 10 min. The collected cells were cultured at 37 °C in RPMI 1640 medium supplemented with 10% (*v*/*v*) FBS, 100 U/mL penicillin, and 100 μg/mL streptomycin for 2 h. The unattached cells were discarded, while the attached PMCs were further cultured and used for pyroptosis assays.

### 4.9. Lentivirus and Retrovirus Packaging

A lentiviral vector expressing Men1 specific shRNA (Men1 RNAi: 5′-GGCAGAAGGTGCACATAGT-3′) was packaged with Lenti-Easy Packaging System in the HEK-293T cells. Retroviral infection was used to overexpress Men1. The recombinant retroviral plasmid pLNCX2-Men1 was co-transfected with helper plasmid pVSVG into the HEK-293T cells. Transfected HEK-293T cells were incubated for 48 h before virus collection.

### 4.10. Transwell Invasion Assay

MLE-12 cells were exposed to 20 μg/mL BLM for 24 h. Cell supernatant was used as a chemoattractant for the Raw264.7 cells, which were infected with retro or lentivirus. The infected cells were trypsinized, resuspended in serum-free DMEM, and then seeded into 8 μm pore-size cell culture inserts. The inserts were incubated with MLE-12 conditional medium (CM) for 16 h. Cells on the upper side of the membrane were wiped out, and cells on the bottom membrane were fixed and stained with crystal violet solution. The migrated cells were photographed with a light microscope and counted in five fields.

### 4.11. Scratch-Wound Healing Assay

MEFs were seeded into 12-well plates and cultured until they reached the monolayer confluency. A scratch was made in the monolayer with a sterile p200 pipette tip. Cell debris was washed with PBS, and then cells were incubated with CM from BLM treated or untreated Raw264.7 cells for 18 h. The scratch area was photographed at 0 and 18 h and analyzed with Image J software. The wound healing ability of MEF was assessed by comparing the scratch area data at 18 and 0 h.

### 4.12. Immunoblotting

Total protein was extracted from cells or crushed tissues with lysis buffer (150 mM NaCl, 10 mM Tris pH 7.2, 5 mM EDTA, 0.1% SDS, 1% sodium deoxycholate, 1% Triton X-100 and protease inhibitors). Sample protein concentrations were determined with a BCA protein assay kit and adjusted for immunoblotting analysis. Protein samples were fractionated by SDS-PAGE and then transferred onto PVDF membranes. The membranes were blocked with 5% BSA in Tris-buffered saline with Tween 20 (TBST) and then incubated with primary antibodies overnight at 4 °C. Next, the membranes were incubated with HRP coupled secondary antibodies, and the results were visualized with ECL luminescent liquid on a Tanon 5200 Multi FluorChem imaging system.

### 4.13. Quantitative Real-Time PCR (qRT-PCR)

Total cell RNA was extracted with TRIzol reagent. A total of 1 μg RNA was reverse transcribed to cDNA using a cDNA Synthesis Kit for qRT-PCR analysis. FastStart Universal SYBR Green Master (ROX) reagent was used to perform qRT-PCR in a StepOnePlus system (Applied Biosystems, Carlsbad, CA, USA). GAPDH was used as a loading control, and the relative mRNA expressions were calculated using the 2–ΔΔCt method. The specific mouse primers were generated by BGI (Beijing, China).

 *GAPDH*:

forward: 5′-GCACCACCAACTGCTTAG-3′,

reverse: 5′-GCAGGGATGATGTTCTGG-3′;

 *TNF-α*:

forward: 5′-AACGCCCTCCTGGCCAA-3′,

reverse: 5′-GCAAATCGGCTGACGGTG-3′;

 *IL-6:*

forward: 5′-CCACTTCACAAGTCGGAGGCTTA-3′,

reverse: 5′-GCAAGTGCATCATCGTTGTTCATAC-3′;

 *Arg-1*:

forward: 5′-CTCCAAGCCAAAGTCCTTAGAG-3′,

reverse: 5′-AGGAGCTGTCATTAGGGACATC-3′;

 *IL-10*:

forward: 5′-AATTCCCTGGGTGAGAAGCTGA-3′,

reverse: 5′-CTCTTCACCTGCTCCACTGCC-3′;

 *IL-1β*:

forward: 5′-GCAACTGTTCCTGAACTCAACT-3′,

reverse: 5′-ATCTTTTGGGGTCCGTCAACT-3′;

 *Col 1a1*:

forward: 5′-GCACGAGTCACACCGGAACT-3′,

reverse: 5′-AAGGGAGCCACATCGATGAT-3′;

 *Fn1*:

forward: 5′-GAAACCTGCTTCAGTGTGTCTG-3′,

reverse: 5′-TTGAATTGCCACCATAAGTCTG-3′;

 *Acta2*:

forward: 5′-CGGGAGAAAATGACCCAGATT-3′,

reverse: 5′-GGACAGCACAGCCTGAATAGC-3′.

### 4.14. Dual-Luciferase Reporter Gene Assay

The potential promoter region of the human *OPN* gene, about two kilobase pairs of length in the 5′-flanking region (−2000~+100 nt), was amplified from the HEK-293T genomic DNA by PCR using the specific primers (forward: 5′- CGCGCTAGCTGGAATTAAGAAAATTGGT-3′, reverse: 5′-CGCAAGCTTACTGAAGCTGTACCTTGGTC-3′). The PCR product was cloned into the luciferase reporter gene vector pGL4.20 and confirmed by DNA sequencing. HEK-293T cells were transfected with *Men1* overexpressing pcDNA-Men1 plasmid, followed by co-transfection with pGL4.20 with or without *OPN* promoter sequence and the renilla vector pGL4.74 in a ratio of 1:50. A total of 24 h later, luciferase activities were determined with a BioTek Synergy HT Multi-Mode microplate reader ((BioTek Instruments, USA). The renilla luciferase activity served as a reference.

### 4.15. Chromatin Immunoprecipitation (ChIP)

The ChIP assay was performed with a ChIP kit following the manufacturer’s instructions. Briefly, Raw264.7 cells transfected with pcDNA-Men1 or pcDNA3.1 were treated with BLM for 24 h, followed by trypsinization and cross-linking with 1.1% formaldehyde. Then, 1.25 M glycine was added to the reaction system to stop cross-linking, and then the cells were lysed with lysis buffer. The cell lysate was sonicated for 3 s with 5 s intervals and 15 cycles to shear the DNA into 200~500 bp fragments. The DNA pieces were immunoprecipitated with either control IgG or menin primary antibodies. The purified DNA was subjected to qRT-PCR. The primers used for the detection of menin binding to *OPN* promoter are as follows:

 P1:

forward: 5′-ACACAAACTCCAGTGGGTGTTG-3′,

reverse: 5′-CTTTGAAGGTCCTTAGCACAC-3′;

 P2:

forward: 5′-GTGACTTGCCCAAGGTCACA-3′,

reverse: 5′-TGGCCATATAGAGCAGAAGAG-3′.

### 4.16. Statistical Analysis

The data are presented as mean ± standard errors of the mean (SEM). Statistic differences were analyzed by one-way ANOVA or Student’s *t*-tests. *p*-values < 0.05 denote significant differences.

## Data Availability

The data in this paper are available from the corresponding author upon reasonable request.

## Supplementary Materials

The following supporting information can be downloaded at: https://www.mdpi.com/article/10.3390/ijms23105385/s1.

## Abbreviations

Arg-1	Arginase 1
AAV	Adeno associated virus
BLM	Bleomycin
ChIP	Chromatin immunoprecipitation
CM	Culture medium
ECM	Extracellular matrix
GSDMD	Gasdermin D
IF	Immunofluorescence
IHC	Immunohistochemistry
IL-10/1β/18	Interleukin 101β/18
IPF	Idiopathic pulmonary fibrosis
MEF	Mouse embryo fibroblasts
Men1	Multiple endocrine neoplasia 1
NLRP3	NLR family pyrin domain containing 3
OPN	Osteopontin
PMC	Peritoneal macrophages
TNF-α	Tumor necrosis factor
α-SMA	Smooth muscle actin α
