# Supplementary material for: Dichotomous Roles of Men1 in Macrophages and Fibroblasts in Bleomycin—Induced Pulmonary Fibrosis
Source: Int J Mol Sci. 2022 May 11;23(10):5385. doi: 10.3390/ijms23105385 (PMC9140697; doi:10.3390/ijms23105385)
Supplement: Supplementary file 1 [file ijms-23-05385-s001.zip › ijms-1646733-supplementary.pdf]

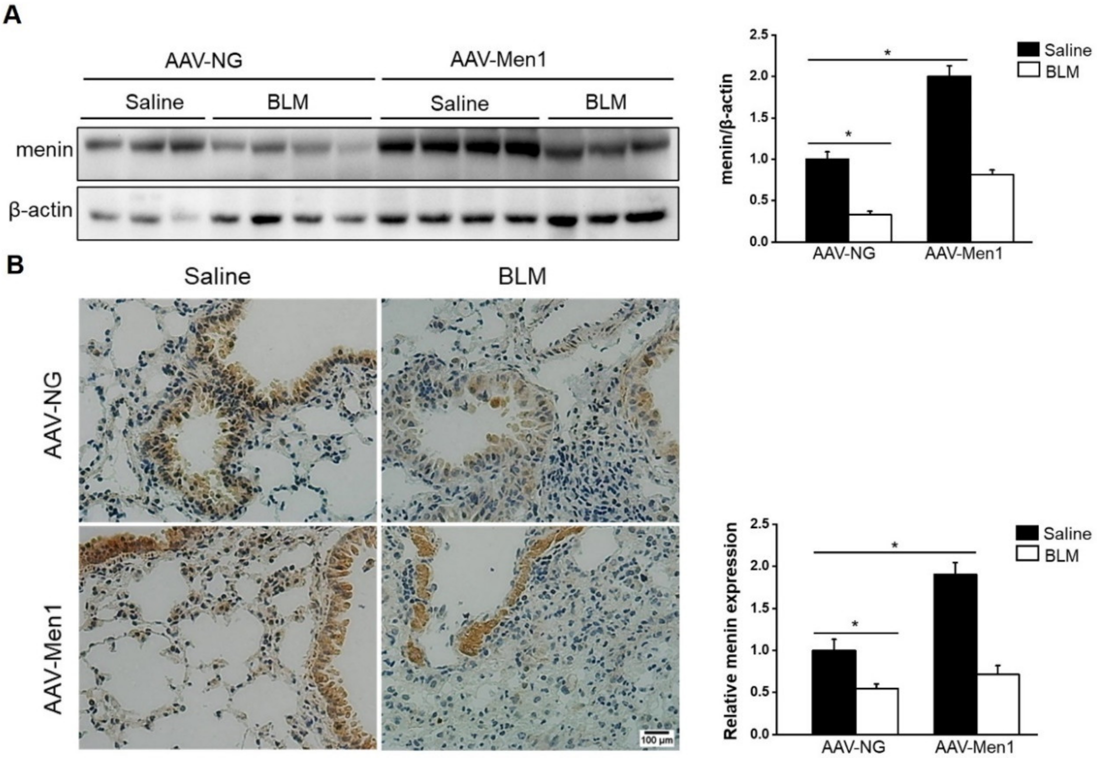

Figure S1. Menin was overexpressed upon AAV-Men1 infection. (**A**) The protein level of menin in lung tissues was determined by Western blotting and the data were quantified by Image J software (n = 7 for each group). (**B**) The expression and distribution of menin were virtualized by IHC staining and the data were quantified by Image J software (*n* = 7 for each group). Scale bar: 100 μm. *, *P*<0.05.

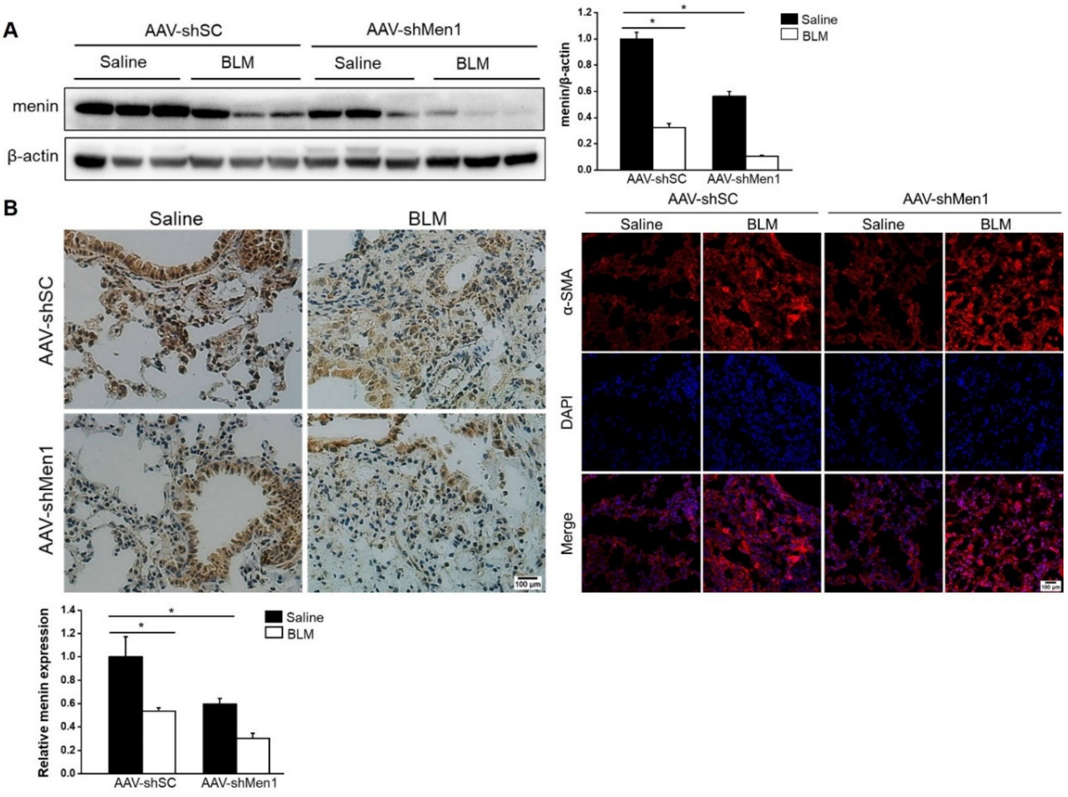

Figure S2. The expression of menin was inhibited by AAV-shMen1 infection. (**A**) The protein level of menin in lung tissues was detected by Western blotting. Data quantification was performed by

Image J software (n = 6 for each group). (**B**) Expression and distribution of menin were determined by IHC staining and data quantification was performed by Image J software (n = 5 for each group). *, P<0.05. (**C**) The expression of α-SMA in lung tissue was visualized with IF staining.α-SMA and the nuclei were labeled with Cy5 and DAPI, respectively. Scale bar: 100 μm.

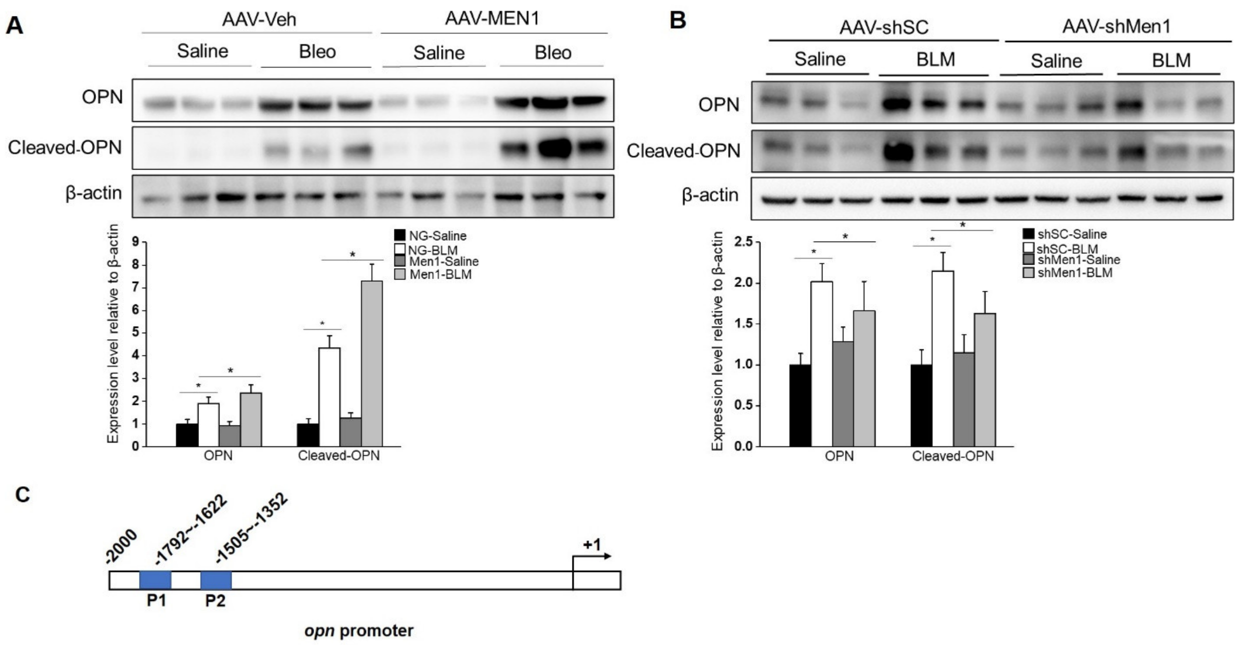

Figure S3. Menin regulates OPN expression by transcriptional way. (**A-B**) The level of OPN protein in lung tissue was determined by Western blotting. (n = 7 for AAV-NG and AAV-Men1 goups, n = 6 for AAV-shSC and AAV-shMen1 groups). (**C**) The specific primers to the OPN promoter region were designed.
